# Associations of Major Dietary Patterns and Dietary Diversity Score with Semen Parameters: A Cross-Sectional Study in Iranian Infertile Men

**DOI:** 10.22074/ijfs.2020.6196

**Published:** 2020-10-12

**Authors:** Masha Shirani, Praveen Saneei, Mehran Nouri, Mohamadreza Maracy, Homayoun Abbasi, Gholamreza Askari

**Affiliations:** 1 Food Security Research Center, Isfahan University of Medical Sciences, Isfahan, Iran; 2 Students' Research Committee, Isfahan University of Medical Sciences, Isfahan, Iran; 3 Department of Community Nutrition, School of Nutrition and Food Science, Isfahan University of Medical Sciences, Isfahan, Iran; 4 Shiraz University of Medical Sciences, Shiraz, Iran; 5 Department of Epidemiology and Biostatistics, School of Public Health, Isfahan University of Medical Sciences, Isfahan, Iran; 6 Isfahan Fertility and Infertility Center, Isfahan, Iran

**Keywords:** Dietary Diversity Score, Dietary Pattern, Infertility, Spermogram

## Abstract

**Background:**

This cross-sectional study pointed to assess the relationship between major dietary patterns and dietary diversity score with semen parameters, in infertile Iranian males.

**Materials and Methods:**

In this cross-sectional study, 260 infertile men (18-55 years old) who met the inclusion criteria, entered the study. Four Semen parameters, namely sperm concentration (SC), total sperm movement (TSM), normal sperm morphology (NSM) and sperm volume were considered according to spermogram. A 168-item food frequency questionnaire (FFQ) was used to collect dietary intakes and calculate dietary diversity score. Factor analy- sis was used to extract dietary patterns.

**Results:**

The following four factors were extracted: “traditional pattern”, “prudent pattern”, “vegetable-based pattern” and “mixed pattern”. After adjusting potential confounders, those in the highest quartile of the traditional pattern had 83% less odds for abnormal concentration, compared with the first quartile (OR=0.17, 95% CI: 0.04-00.73); however, subjects in the highest quartile of this pattern had 2.69 fold higher odds for abnormal sperm volume as compared with those of the first quartile (95%Cl: 1.06-6.82). Men in the second quartile of prudent pattern had 4.36 higher odds of an abnormal sperm volume in comparison to the reference category (95%CI: 1.75-10.86), after considering potential confounders. With regard to mixed pattern, men in the second, third and fourth quartile of this pattern had respectively 85 (5%Cl: 0.03-0.76,), 86 (95%Cl: 0.02-0.75) and 83 % (95%Cl: 0.034-0.9) less odds of abnormal concentration, compared with the first quartile. Additionally, no significant association was found between dietary diversity score and sperm quality parameters.

**Conclusion:**

Higher intake of the traditional diet was linked to lower abnormal semen concentration and poorer sperm volume. Also, the mixed diet was associated with reduced prevalence of abnormal semen concentration.

## Introduction

Infertility is described as inability of a couple to conceive within 12 months or more of regular unprotected intercourse (1). It has been a global issue and a clinical problem in recent decades affecting 15% of all couples in reproductive ages (2). A meta-analysis reported a 10.9% infertility rate in Iranian population. In approximately 40% of infertile couples, male factors are the only or a contributing reason in the inability to have a successful pregnancy(3).

Some disorders such as varicocele, anatomical or hormonal problems, genetic anomalies and infections were shown to contribute to male infertility. Moreover, circumferential factors such as air pollution, industrial chemicals, depression, alcohol use and smoking have been considered potential risk factors reducing sperm quality parameters in developed countries (4). Studies have suggested associations between semen quality and lifestyle factors, including physical activity and dietary intakes(5). According to results of human and animal studies, direct correlations exist between reactive oxygen species (ROS) production in spermatozoa and antioxidant status (6).

However, several studies focused on associations between food components, such as antioxidants and semen quality , and analysis of dietary pattern revealed a new and comprehensive outlook for assessment of the association between diet and the chance of chronic diseases (7). This approach has examined the influences of whole diet, instead of considering individual nutrients or foods, and could be more useful for prediction of risk factors (8). In Thai men, a western pattern was correlated with poorer sperm density and normal sperm morphology while a healthy pattern was not related to any sperm quality parameters (9). Also, a prudent pattern was correlated with a reduced risk of asthenozoospermia in Iranian males while the western pattern was related with increased odds of asthenozoospermia(10).

Dietary diversity score (DDS) is another index to evaluate the quality of diet. Recent researches suggested that, more various diets could be protective against some chronic diseases such as cancers or cardiovascular diseases. A varied diet is associated with higher intake of micronutrients and macronutrients such as fiber, vitamins and calcium, all of which being negatively associated with obesity(11). As we know, the linkage between dietary diversity score and infertility has not been studied. Furthermore, the findings of previous investigations were controversial; so, more researches especially in Middle Eastern populations are needed. It is worth noting that unlike other subfertility risk factors, diet is an adjustable factor and can be considered in counseling of infertile men. Therefore, this study was designed to assess the association of premier dietary patterns and dietary diversity score with semen parameters, in Iranian infertile male.

## Materials and Methods

### Participants

This cross-sectional study was performed in 2018 in Isfahan, a central province in Iran. In total, 270 participants who referred to a major infertility clinic, with primary or secondary history of infertility and aged between 18-55 years old, were selected. Before entering the study, all patients signed the consent letter. Those with history of disorders (infection, azoospermia, genital surgery or other genital diseases, anatomical disorders, or endocrinopathy), or metabolic diseases, or those receiving hormone therapy, supplements, cytotoxic drugs or immunosuppressant were not included in the study. Also, patients with history of psychiatric or physiological disease that could affect sperm quality, were not included (12). Furthermore, 10 participants with incomplete information or caloric intakes more than 4200 kcal/day were excluded from the study. Finally, 260 subjects who met the inclusion criteria entered the study. The study was ethically approved by Ethics committee of Isfahan University of Medical Sciences (IUMS), Isfahan, Iran (No.397387).

### Assessment of semen parameters

After three days of abstinence, semen samples were obtained. The samples were collected into sterile containers and liquefied at 37°C for 30 minutes before analysis. Samples were analyzed according to the 5^th^ edition of World Health Organization (WHO) laboratory manual for the evaluation of human semen. Four semen parameters including sperm concentration (SC), total sperm movement (TSM), normal sperm morphology (NSM) and sperm volume, were assessed. Evaluation of the motility of sperm was done based on WHO criteria and the motility was scored from A to D. A+B is defined as total progressive motility, C is defined as non-progressive motility, A+B+C is defined as total motility and D is defined as immotile sperm (13).

### Assessment of dietary intakes

A validated 168-item food frequency questionnaire (FFQ) was used to evaluate dietary intakes of the individuals. This questionnaire has previously been shown to be valid for evaluating Iranian food intakes (14). All participants noted their average consumption of each food item in terms of the specified serving size over the past year. The speciﬁc categories were: 6 times or more a day, 3-5 times a day, 2-3 times a day, every day, 5-6 times /week, 2-4 times /week, once a week, 1-3 times a month and less than once a month. Nutritionist IV software, which is modified for Iranian meals, was used to extract dietary intakes of participants based on their FFQ.

### Dietary diversity score

In this study, DDS was calculated according to the study by Kant et al.(15). In this method, food items are categorized into 5 categories: 1. bread and cereals, 2. vegetables, 3. fruits, 4. meat and substitutes, and 5. dairy products. Then, each group was divided into several subgroups. Bread and cereals were divided into seven subgroups: white bread and refined grains, whole bread branny biscuits, macaroni, breakfast cereals, rice and flour. Vegetables were classified into seven groups: fines herbs, potato, starchy vegetables, tomato, yellow vegetables, green vegetables and legumes. Fruits consisted of berries and citrus fruits, and other fruits. Meat and substitutes were divided into four subgroups including: red meat, poultry, sea food and egg. Dairy products included three groups: milk, yogurt, cheese and dried whey. Each group was given a score from 0 to 2, so the maximum score was 10. To calculate the score for each group, the whole subgroups consumed by a person were divided by total subgroups and then multiplied by 2. For example, if a participant consumed four subgroups of cereals, the score was calculated as(4÷7)×2=1.14.

### Assessment of other variables

A structured questionnaire was used to collect other demographic data, medical history, alcohol or cigarette use and supplements intake. Subjects were interviewed faceto- face. Weight (with accuracy of 0.1 kg) and height (with accuracy of 0.5 cm) were measured. Body mass index (BMI) was then calculated in kilograms per meter square.

### Statistical analysis

Continuous variables are reported as mean (± SD or SE). The normality of the data was assessed by using the Shapiro–Wilk test. In this study, we categorized the 168 food items into 32 food groups to facilitate the analysis. The classification was based on the nutrient profiles resemblance, or recipe of foods (10, 16). Major dietary factors were identified by using factor analysis with varimax rotation method. Considering the Eigen value >1.9 and scree plot, four factors were selected.

We applied the quartiles of major dietary patterns or DDS scores to assess the relationships between dietary intakes and sperm parameters. One-way analysis of variance (ANOVA) (or Kruskal Wallis test as a non-parametric test) or chi-square tests were used to assess general characteristics and dietary intakes of study participants across different quartiles of dietary intakes. Differences in the sperm parameters versus food intake amounts were compared using chi-square test. Abnormal semen parameters were defined as Oligospermia : SC<20 M/ml, TSM<60%, sperm volume<3 ml and NSM<65% (12). As we were not able to analyze data with NSM<65% in our study, NSM<4% as the world health organization (WHO) cut point was considered for normal morphology (13). The frequencies of abnormal semen quality parameters in each quartile, were compared by the chi-square test or fisher exact test if required. Multiple logistic regression [odds ratios (ORs) with 95% confi dence interval (CI)] was used to assess the relationship between major dietary patterns and DDS and sperm quality parameters. In adjusted model, potential confounding variables including age, BMI, education, total energy intake, alcohol use, smoking and vitamin-mineral use were justified by analysis of covariance (ANCOVA). In all models, the first quartile was considered the reference level. For all analyses, SPSS software (Version 25) was used and significance level was considered P<0.05.

## Results

Based on Eigen values and scree plot, we selected four factors. Factor-loading matrixes of foods and food group classification are provided in Table 1. A positive loading in a factor showed a linear association with the factor, while a negative loading shows that the food group was reversely correlated with the factor. The “traditional pattern” was defined by high intakes of organ meat, dairy products, saturated fats, fruits, fruit juice, legumes, sugary beverages, deserts and sweets. The “prudent pattern” was defined by high intakes of nuts, olive oil, red meat, dried fruits, fruit juice, fish and low intake of refined grains. The “vegetable-based” pattern was defined by high intakes of fruits, leafy vegetables, yellow vegetables, legumes, tomato and other non-starchy vegetables. The “mixed pattern” was defined by high intakes of green/black tea, vegetable oils, and potato, and low intake of whole grains and soy bean. Each participant was given scores for the traditional, prudent, vegetable-based and mixed major dietary patterns according to his/her consumption of items.

**Table 1:** Loadings of foods and food groups across major dietary patterns


	Component			
	1	2	3	4

Sugary beverage	0.659 ^*^			
Organ meat	0.640			
Processed food	0.627			
Pickles	0.614			
Sauce	0.613			
Saturated fat	0.596			
Dairy products	0.521			
Fruits	0.411		0.523	
Sweets and deserts	0.512	0.446		
Snacks	0432	0.398		
Red meat		0.696		
Dried fruits		0.793		
Olive oil		0.679		
Refined grains		-0. 525		0.309
Nuts		0.511		
Fruit juice	0.422	0.475		
Fish		0.397		
Poultry			0.450	
Legumes	0.305		0.449	
Leafy vegetables		0.319	0.564	
Yellow vegetables			0.438	
Tomato			0.678	
Non starchy vegetables			0.615	
Cheese			0.428	
Skim fat dairy			0.349	
Egg			0.335	
Potato	0.345			0.403
Salt				0.625
Vegetable oils				0.505
Tea(green/black)			0.398	0.480
Soy bean				-0.385
Whole grain				-0.325


Factor loadings less than 0.3 were omitted for simplicity. Principal Component Analysis as the extraction method and Varimax with Kaiser Normalization as the rotation method, were applied. 1, 2, 3, 4; These numbers show four categories of food patterns.

Mean age, BMI and TEE of study participants and semen quality parameters are shown in Table 2. Demographic data showed that 36.5% of participants smoked cigarette and 20.3% used alcoholic drinks. Additionally, the education of 22.6% of participants was less than diploma. Dietary intakes of selected nutrients and energy intake of study participants among different quartiles of dietary patterns, are reported in Table 3. Intakes of energy (P=0.01), protein (P=0.002), total fiber (P<0.001), eicosapentaenoic acid (EPA) (P=0.008), docosahexaenoic acid (DHA) (P=0.002), folate (P<0.001), vitamin E (P=0.001) and selenium (P<0.001) were significantly different across quartiles of traditional pattern. General characteristics and energy intake of individuals among different quartiles of DDS are shown in Table 4. The distribution of energy intake (P<0.001) and BMI (P=0.018) was different among quartiles of DDS.

To clarify major dietary patterns-semen quality relation or DDS-semen quality relation, prevalence of abnormal sperm parameters in quartiles of different dietary patterns or DDS was evaluated (Table 5). The prevalence of abnormal SC was lower in the top quartile of traditional dietary intake, compared with the bottom quartile (P=0.015). Also, the abnormal SC prevalence was reduced in the second quartile of mixed diet in comparison to the first category (P=0.041). Moreover, a significant difference in terms of abnormal sperm volume prevalence was found across categories of prudent dietary intake (P=0.035). However, there was no significant relationship between different quartiles of DDS and prevalence of abnormal semen parameters.

Multivariable- adjusted odds ratio for abnormal semen quality across quartiles of premier dietary patterns and DDS, are shown in Table 6. After adjusting potential confounders, those in the highest quartile of the traditional dietary pattern, odds were 83% less for abnormal SC, compared with the first quartile (OR=0.17, 95% confidence interval (95% CI); 0.04-00.73); however, subjects with the highest quartile of this pattern had a 2.69 fold higher odds for abnormal sperm volume as compared with the first quartile (95% Cl: 1.06-6.82). Men in the second quartile of prudent dietary pattern had 4.36 higher odds of an abnormal sperm volume in comparison to the reference category (95% CI: 1.75- 10.86), after considering potential confounding variables. With regard to mixed dietary pattern, men in the second, third and fourth quartile of this pattern had respectively 85 (95% Cl: 0.03-0.76), 86 (95% Cl: 0.02-0.75) and 83 % (95% Cl: 0.034-0.9) less odds for abnormal SC, compared with the first quartile.

Furthermore, there was no significant relationship between DDS and semen parameters.

**Table 2:** Characteristics of participants


Characteristics	Mean ± SD

Age(Y)	31.24 ± 4.33
BMI (Kg/m^2^)	26.94 ± 4.09
Energy (Kcal)	2516.51 ± 686.94
MET (MET-h/week) Sperm parameters	29.2 ± 2.12
Density (Mol/ml)	13.11 ± 16.01
Volume(ml)	4.13 ± 2.06
Total motility (%)	29.76 ± 18.08
Normal. Morphology (%)	4.23 ± 10.68
DDS	5.09 ± 1.29


SD; Standard deviations, BMI; Body mass index, and DDS; Dietary diversity score as assessed by Analysis of variance (n=260).

**Table 3:** Dietary intakes of energy and selected nutrients of study participants among different quartiles of dietary patterns


	Traditional			Prudent			Vegetable based			Mixed		
	Q1	Q4	P	Q1	Q4	P	Q1	Q4	P	Q1	Q4	P

Energy (Kcal/d)	2306 ± 678	2622 ± 605	0.01	2630 ± 732	2482 ± 658	0.39	2501 ± 612	2427.79 ± 654	0.40	2450 ± 672	2481.06 ± 631	0.42
Proteins (% of energy)	15.90 ± 2.40	14.30 ± 2.10	0.002	14.67 ± 2.44	15.28 ± 2.76	0.44	15.79 ± 2.42	14.76 ± 2.66	0.01	15.53 ± 2.49	15.02 ± 2.56	0.42
Fats (% of energy)	29.10 ± 6.40	29.70 ± 5.30	0.06	30.13 ± 5.77	31.07 ± 6.82	0.23	29.54 ± 5.84	30.72 ± 6.18	0.37	30.67 ± 6.49	29.11 ± 5.74	0.09
Carbohydrates (% of energy)	58.55 ± 8.09	59 ± 5.60	0.06	58.03 ± 6.50	57.35 ± 7.02	0.38	57.97 ± 6.95	58.01 ± 7.34	0.86	57.22 ± 7.83	58.86 ± 7.48	0.39
Total fiber (g/d)	34.30 ± 11.30	48.80 ± 2.10	˂0.001	44.66 ± 17.45	39.61 ± 14.64	0.19	41.62 ± 16.98	38.46 ± 15.40	0.57	37.14 ± 14.61	43.30 ± 17.39	0.17
DHA (mg/d)	0.23 ± 0.21	0.13 ± 0.12	0.002	0.18 ± 0.17	0.22 ± 0.26	0.60	0.27 ± 0.27	0.18 ± 0.17	0.05	0.23 ± 00.24	0.20 ± 0.21	0.56
EPA (mg/d)	0.07 ± 0.07	0.03 ± 0.04	0.008	0.05 ± 0.05	0.06 ± 0.08	0.55	0.08 ± 0.09	0.05 ± 0.05	0.07	0.07 ± 0.08	0.05 ± 0.06	0.53
SFA (g/d)	26.07 ± 11.50	27.12 ± 8.80	0.38	28.05 ± 9.85	37.35 ± 12.59	0.28	26.39 ± 9.11	26.99 ± 10.46	0.30	28.19 ± 11.13	25.94 ± 10.31	0.42
Folate (mg/d)	518 ± 117	613 ± 163	˂0.001	593.23 ± 177.16	541.35 ± 118.73	0.15	574.47 ± 159.23	532.66 ± 131.85	0.37	530.88 ± 111.85	567.90 ± 147.01	0.40
Zinc (g/d)	13.50 ± 4.60	14 ± 4.10	0.34	14.58 ± 4.86	14.14 ± 4.20	0.79	14.35 ± 4.45	13.53 ± 4.15	0.58	14.43 ± 4.85	13.45 ± 3.81	0.37
Selenium (g/d)	97.40 ± 28.60	131 ± 54	˂0.001	131.43 ± 52.68	114.08 ± 38.67	0.01	118.48 ± 51.67	111.99 ± 38.90	0.80	102.10 ± 26.89	118.11 ± 52.18	0.02
Vitamin C (mg/d)	228 ± 134	242 ± 139	0.73	237.51 ± 138.1	244.23 ± 125.60	0.97	248.45 ± 112.62	237.71 ± 141.08	0.92	245.35 ± 147.06	241.65 ± 136.93	0.96
Vitamin E (IU)	10.70 ± 5.00	14.70 ± 5.70	0.001	14.19 ± 5.70	13.67 ± 6.32	0.41	12.62 ± 4.54	13.34 ± 5.72	0.12	12.46 ± 5.45	12.54 ± 4.41	0.01


All data presented as means ± SE. SE; Standard error, P; P value as assessed by analysis of variance (ANOVA) test, DHA; Docosahexaenoic acid, EPA; Eicosapentaenoic acid, SFA; Saturated fatty acids (n=260), Q1; first quartile of intake, and Q4; fourth quartile of intake.

**Table 4:** Characteristics of participants across quartile of dietary diversity score (DDS


	Q1 (˂4.2) (n=66)	Q2 (4.2-5.15) (n=64)	Q3 (5.16-5.9)(n=68)	Q4 (>5.9)(n=62)	P

Age (Y)	30.6 ± 3.70	31.16 ± 3.55	30.7 ± 4.39	32.53 ± 5.33	0.142^a^
PA (MET/h)	29.63 ± 2.03	29.23 ± 2.28	28.92 ± 2.24	29.24 ± 1.84	0.421^b^
WC (cm)	92.95 ± 8.56	93.63 ± 10.61	95.50 ± 9.74	96.01 ± 12.25	0.221^a^
BMI (kg/m^2^)	25.87 ± 3.38	26.54 ± 3.82	27.39 ± 4.32	27.98 ± 4.53	0.018^b, c^
Energy intake (kcal)	2175 ± 578.10	2370 ± 610.30	2620 ± 599.80	2916 ± 737.6	<0.001^a, d^


All data presented as means ± SD. SD; Standard deviations, P; P value: ^a^; Assessed by Kruskal-Wallis test, ^b^; Assessed by ANOVA test, ^c^; Body mass index (BMI) was significantly different between the first and forth quartile of DDS (P=0.018),^ d^; The distribution of energy intake was different between the first and third, first and fourth, second and fourth quartile of DDS (P<0.001), PA; Physical activity, and WC; Waist circumference.

**Table 5:** The associations of abnormal semen quality with dietary patterns and DDS


Quartiles		Concentration (˂20 M/ml versus ≥20)	Total motility (˂60% versus≥60)	Normal morphology (˂4% versus≥4)	Volume (<3 ml versus≥3)

Traditional diet	Q1	93.8	92.3	83.1	30.8
	Q2	92.3	95.4	80	21.5
	Q3	84.6	93.8	75.4	29.2
	Q4	76.9	95.4	80	33.8
	P	0.015	0.85	0.75	0.45
Prudent diet	Q1	81.5	96.9	84.6	21.5
	Q2	89.2	92.3	84.6	41.5
	Q3	86.2	96.9	76.9	30.8
	Q4	90.8	90.8	72.3	21.5
	P	0.41	0.30	0.21	0.035
Vegetable-based	Q1	90.8	93.8	83.1	21.3
	Q2	89.2	95.5	75.4	32.3
	Q3	83.1	89.2	83.1	32.3
	Q4	84.6	95.4	76.9	27.7
	P	0.51	0.15	0.57	0.60
Mixed Diet	Q1	96.5	93.8	81.5	27.7
	Q2	81.5	95.4	81.5	32.3
	Q3	83.1	90.8	81.5	32.3
	Q4	86.2	96.9	73.8	23.1
	P	0.041	0.48	0.62	0.60
DDS	Q1	89.4	97	80.3	28.8
	Q2	87.5	92.2	78.1	34.4
	Q3	86.8	95.6	88.2	26.5
	Q4	83.8	91.9	71	25.8
	P	0.83	0.52	0.10	0.70


All values are presented by percentage, P; P value as assessed by chi square test, DDS; Dietary diversity score (n=260), Q1; first quartile of intake, Q2; second quartile of intake, Q3; third quartile of intake, and Q4; fourth quartile of intake.

**Table 6:** Multivariable- adjusted odds ratio for abnormal semen quality across quartiles of major dietary patterns and DDS


	Concentration˂20 M/ml	P	Total motility˂60%	P	Normalmorphology˂4%	P	Volume˂3 ml	P

Traditional dietary pattern									
Crude	Q1	Reference		Reference		Reference		Reference	
	Q2	0.71 (0.17-2.97)	0.64	2.17(0.43-10.78)	0.34	0.83(032-2.14)	0.70	0.78 (0.33-1.84)	0.58
	Q3	0.31 (0.08-1.17)	0.08	1.26(0.27-5.88)	0.76	0.62(0.24-1.58)	0.32	1.42 (0.62-3.26)	0.40
	Q4	0.16 (0.04-0.66)	0.01	1.57(0.28-8.84)	0.60	0.80(0.29-2.24)	0.68	2.33 (0.95-5.72)	0.06
Adjusted model	Q1	Reference		Reference		Reference		Reference	
	Q2	0.76 (0.17-3.36)	0.72	1.96 (0.35-10.96)	0.44	1.26(0.53-2.95)	0.59	0.79 (0.3-1.89)	0.6
	Q3	0.33 (0.08-1.32)	0.11	3.44 (0.50-23.25)	0.20	0.7(0.3-1.60)	0.04	1.65 (0.69-3.92)	0.25
	Q4	0.17 (0.04-0.73)	0.01	3.35 (0.44-25.43)	0.24	1.08(0.44-2.67)	0.85	2.69 (1.06-6.82)	0.03
Prudent dietary pattern									
Crude	Q1	Reference		Reference		Reference		Reference	
	Q2	0.77 (0.23-2.54)	0.67	0.38(0.06-2.53)	0.32	1.01(0.35-2.86)	0.98	4.45 (1.80-10.96)	0.001
	Q3	0.70 (0.24-2.05)	0.51	0.97(0.12-7.70)	0.98	0.59(0.23-1.56)	0.29	2.50 (1.04-6.01)	0.03
	Q4	1.97 (0.65-5.96)	0.22	0.25(0.04-1.34)	0.10	0.51(0.21-1.26)	0.14	1.20 (0.50-2.85)	0.68
Adjusted model	Q1	Reference		Reference		Reference		Reference	
	Q2	0.69 (0.2-2.38)	0.56	0.27 (0.03-2.36)	0.24	1.18(0.5-2.82)	0.69	4.36 (1.75-10.86)	0.002
	Q3	0.79 (0.24-2.57)	0.69	0.80 (0.07-8.81)	0.85	1.13(0.49-2.60)	0.76	2.39 (0.97-5.92)	0.05
	Q4	1.89 (0.59-6.08)	0.28	0.17 (0.02-1.18)	0.07	1.18(0.54-2.59)	0.66	1.13 (0.46-2.75)	0.77
Vegetable-based dietary pattern									
Crude	Q1	Reference		Reference		Reference		Reference	
	Q2	0.83 (0.24-2.87)	0.77	4.95 (0.50-48.28)	0.16	0.60 (0.24-1.48)	0.27	1.68 (0.74-3.82)	0.21
	Q3	0.50 (0.15-1.59)	0.24	0.61 (0.14-2.56)	0.50	0.90 (0.34-2.37)	0.84	1.69 (0.73-3.89)	0.21
	Q4	0.51 (0.15-1.65)	0.26	1.36 (0.27-6.63)	0.70	0.64 (0.26-1.56)	0.33	1.29 (0.56-2.95)	0.53
Adjusted model	Q1	Reference		Reference		Reference		Reference	
	Q2	0.83 (0.23-2.95)	0.77	3.73 (0.34-40.03)	0.27	0.75 (0.33-1.71)	0.50	1.69 (0.73-3.87)	0.21
	Q3	0.54 (0.16-1.83)	0.32	0.61 (0.11-3.25)	0.56	0.59 (0.26-1.34)	0.21	1.78 (0.75-4.19)	0.18
	Q4	0.49 (0.14-1.66	0.25	2.15 (0.34-13.31)	0. 40	0.73 (0.32-1.65)	0.45	1.14 (0.49-2.67)	0.74
Mixed dietary pattern									
Crude	Q1	Reference		Reference		Reference		Reference	
	Q2	0.15 (0.03-0.77)	0.02	1.28 (0.25-6.49)	0.76	1.16 (0.45-2.94)	0.75	1.26 (0.56-2.84)	0.56
	Q3	0.15 (0.03-0.74)	0.02	0.69 (0.15-3.10)	0.63	1.02 (0.40-2.59)	0.96	1.16 (0.52-2.62)	0.70
	Q4	0.17 (0.03-0.89)	0.03	2.22 (0.37-13.34)	0.38	0.68 (0.28-1.61)	0.38	0.69 (0.30-1.59)	0.39
Adjusted model	Q1	Reference		Reference		Reference		Reference	
	Q2	0.15 (0.03-0.76)	0.02	2.27 (0.33-15.51)	0.4	0.87 (0.39-1.89)	0.72	1.29 (0.56-2.98)	0.53
	Q3	0.14 (0.02-0.75)	0.02	1.10 (0.17-6.92)	0.91	1.58 (0.70-3.56)	0.26	1.22 (0.53-2.79)	0.62
	Q4	0.17 (0.034-0.9)	0.03	3.43 (0.44-26.64)	0.23	1.74 (0.78-3.91)	0.17	0.73 (0.31-1.69)	0.46
DDS									
Crude	Q1	Reference		Reference		Reference		Reference	
	Q2	0.83 (0.28-2.44)	0.73	0.36 (0.06-1.97)	0.24	0.72 (0.34-1.50)	0.38	1.29 (0.61-2.72)	0.49
	Q3	0.77 (0.27-2.22)	0.63	0.67 (0.10-4.18)	0.67	1.31 (0.60-284)	0.48	0.89 (0.41-1.90)	0.76
	Q4	0.61 (0.21-1.73)	0.36	0.35 (0.06-1.90)	0.22	0.78 (0.37-1.66)	0.53	0.86 (0.39-1.87)	0.70
Adjusted model	Q1	Reference		Reference		Reference		Reference	
	Q2	0.75 (0.24-2.31)	0.62	0.4 (0.07-2.37)	0.31	0.83 (0.34-1.98)	0.67	1.43 (0.66-3.08)	0.35
	Q3	0.82 (0.26-2.52)	0.73	1.1 (0.15-7.76)	0.91	1.56 (0.57-4.27)	0.38	1.01 (0.44-2.27)	0.98
	Q4	0.57 (0.17-1.90)	0.36	0.99 (0.13-7.33)	0.99	0.52 (0.21-1.32)	0.17	1.04 (0.43-2.49)	0.90


All data presented as means ± SD. SD; Standard deviations, P; P value: ^a^; Assessed by Kruskal-Wallis test, ^b^; Assessed by ANOVA test, ^c^; Body mass index (BMI) was significantly different between the first and forth quartile of dietary diversity score (DDS, P=0.018), ^d^; The distribution of energy intake was different between the first and third, first and fourth, second and fourth quartile of DDS (P<0.001), PA; Physical activity, and WC; Waist circumference.

## Discussion

We found that a higher traditional diet intake was correlated with reduced abnormal sperm concentration and poorer sperm volume in Iranian infertile men. Furthermore, the mixed diet showed a significant relationship with lower levels of abnormal sperm concentration. The novelty of our study was the evaluation of the relationship between DDS and sperm quality parameters in infertile men, even though there was no significant association between DDS and sperm quality parameters in our study.

The findings of some recent studies on the association between diet and sperm parameters agreed with the present study while some others did not. One study was conducted among sub-fertile men referring to an in vitro fertilization clinic in the Netherlands; in this study, the “health-conscious” dietary pattern and the “traditional Dutch” pattern were extracted. There was a reverse association between the health-conscious diet and DNA fragmentation index, while the traditional diet, as seen in our study, was positively related with sperm concentration and folate level in red blood cells. Nevertheless, the authors did not find any association between dietary patterns and sperm movement (17). Another study done at the University of Rochester on healthy men, indicated that prudent pattern was only related with percentage of sperm with progressive motility, while the Western pattern was not correlated with any sperm quality parameters (18). A systematic review and meta-analysis of six observational studies on 8207 participants, declared that individuals with the highest adherence to healthy dietary pattern versus those with the lowest adherence, had significantly higher level of sperm concentration. However, in this analysis, there was no significant association between eating dietary patterns and other sperm parameters (19) . Another research done in Poland, suggested that a pro-healthy pattern was not related with any sperm quality parameters. Similarly, in our study, the prudent dietary pattern was only related to sperm volume (20).

In the present study, the traditional dietary pattern was defined by high intakes of dairy products, saturated fats, fruits, sugary beverages and sweets. Animal products such as meat and dairy products are major sources of protein and micronutrients. Trans fatty acids (TFAs), saturated fatty acids (SFAs) and preservative agents or hormonal residues like xenobiotics or anabolic steroids, may affect sperm quality (21). Previous investigations showed that total dairy food intake was reversely associated with NSM and among physically active young men, whole-fat dairy intake was related to a significantly lower PRM (progressive motility), whereas intake of low-fat milk was specifically related to a higher progressive motility and sperm concentration. Consumption of low-fat and skimmed milk was also related with higher levels of insulin and insulin-like growth factor 1 (IGF-1) (22). With regard to dietary fat food intakes, fat-rich foods, such as hydrogenated fat and saturated fat, might also reduce the sperm quality in humans (21). Based on the results of a systematic review of 17 randomized trials, antioxidant supplementation improved sperm movement in most trials. Additionally, there are some important minerals with antioxidant role such as zinc, selenium and vitamin E ,that can be received via diet instead of supplements(23, 24). In our study, intakes of folate, selenium and vitamin E were significantly higher in top level of traditional diet. Furthermore, fruits and vegetables, which are the main source of fi ber intake, can directly bind to unconjugated estrogens and reduce the estrogen level of plasma.

Additionally, the mixed diet was significantly related to lower abnormal SC; this might be possibly due to the presence of catechins and the afl avins in green tea (GT) and black tea (BT) (components of mixed diet), respectively. These bioactive phytochemicals could be related to the antioxidant activity (25). Low intake of SFAs or TFAs in vegetable oils as well as low intake of soy bean in this dietary pattern, could be responsible for the observed associations. High concentration of phytoestrogens in soy foods can be responsible for their negative effects on male fertility. Phytoestrogens are known to have destructive effects on the male endocrine system with unfavorable effects on fertility. The results of a study on Caucasian subjects showed that lower sperm concentration was related to a higher intake of soy foods (26).

Surprisingly, a vegetable-based pattern mostly including fruits and vegetables, was not related to any sperm quality parameters, which is in contrast with the findings of some previous studies. However, fruits and vegetables contain large amounts of some minerals such as selenium, vitamin C and vitamin E, which may indirectly improve semen quality through their anti-inflammatory and protective role against free radicals. The presence of environmental contaminants including chlorinated pollutants and pesticides might be a possible explanation for this observation. Therefore, the antioxidative role of fruits and vegetables could be diminished by possible toxic effects of pollutants and pesticides (27).

DDS is an index to evaluate the quality of diet. Moreover, it can represent intake of micronutrients or energy. A previous study among Tehranian women showed that increasing diversity score in cereals was related to higher intake of carbohydrates, proteins and calcium; however, increasing fruits and vegetables scores were related to higher intake of vitamin A and C and lower intake of energy(28). Therefore, it is important to note that intake of which food groups increases the DDS.

Strengths of this study included the use of dietary pattern analysis, instead of nutrient or whole food analysis, which more closely reflects overall diet and interaction between all components and the ability to adjust multiple potential confounders (29). Some limitations should be noted while interpreting the results of the study. The design of the study was the main one as determining the direction of association in cross-sectional studies is impossible. Another limitation of the study was the use of FFQ to evaluate habitual dietary intake. Although a validated FFQ with adequate validity and reproducibility was used, it could be prone to measurement error, which usually leads to debilitation of the associations of interest.

## Conclusion

Higher intake of the traditional diet was linked to a lower abnormal semen concentration and poorer sperm volume. Also, the mixed diet was associated to reduced prevalence of abnormal semen concentration. Because of changes in food availability and variation in eating patterns among different socioeconomic status, ethnic groups and cultures, more prospective investigations are needed to explain the correlation between dietary habits and infertility.
